# Molecular Biology of KSHV Lytic Reactivation

**DOI:** 10.3390/v7010116

**Published:** 2015-01-14

**Authors:** Pravinkumar Purushothaman, Timsy Uppal, Subhash C. Verma

**Affiliations:** Department of Microbiology and Immunology, University of Nevada, Reno, School of Medicine, 1664 N Virginia Street, MS 320, Reno, NV 89557, USA; E-Mails: pravinp@medicine.nevada.edu (P.P.); tuppal@medicine.nevada.edu (T.U.)

**Keywords:** KSHV, reactivation, Lytic DNA replication, hypoxia, RTA, LANA

## Abstract

Kaposi’s sarcoma-associated herpesvirus (KSHV) primarily persists as a latent episome in infected cells. During latent infection, only a limited number of viral genes are expressed that help to maintain the viral episome and prevent lytic reactivation. The latent KSHV genome persists as a highly ordered chromatin structure with bivalent chromatin marks at the promoter-regulatory region of the major immediate-early gene promoter. Various stimuli can induce chromatin modifications to an active euchromatic epigenetic mark, leading to the expression of genes required for the transition from the latent to the lytic phase of KSHV life cycle. Enhanced replication and transcription activator (RTA) gene expression triggers a cascade of events, resulting in the modulation of various cellular pathways to support viral DNA synthesis. RTA also binds to the origin of lytic DNA replication to recruit viral, as well as cellular, proteins for the initiation of the lytic DNA replication of KSHV. In this review we will discuss some of the pivotal genetic and epigenetic factors that control KSHV reactivation from the transcriptionally restricted latent program.

## 1. Introduction

Kaposi’s sarcoma-associated herpesvirus (KSHV) or human herpesvirus 8 (HHV 8) is one of the seven human oncogenic viruses, and is the etiologic agent of Kaposi’s sarcoma (KS, a multifocal, angiogenic and inflammatory malignancy of endothelial cell origin), as well as certain B-cell lymphomas, including primary effusion lymphoma (PEL) and multicentric Castleman’s disease (MCD) [1,2,3]. KSHV has been consistently detected in all four clinical forms of KS, including: classical KS, endemic KS in Africa, epidemic AIDS-related KS, and iatrogenic/organ-transplant KS. Lately, a newly characterized KSHV-associated condition, abbreviated as KICS (KSHV Inflammatory Cytokine Syndrome) has been reported in patients with HIV and KSHV co-infection, displaying elevated levels of interleukin-6 (IL-6) production [4]. In healthy seropositive individuals, KSHV causes persistent infection by establishing latency in CD19+ peripheral B-lymphocytes [5].

Since its initial discovery, there has been intense research on understanding the molecular biology of KSHV-mediated tumorigenesis [6]. KSHV is a γ2-lymphotropic-oncogenic-herpesvirus and is genetically linked to the Epstein-Barr virus (EBV), murine γ-herpesvirus-68 (MHV-68), and herpesvirus saimiri (HVS) (reviewed in [7]). Similar to the other members of herpesvirus family, KSHV enters the host cell as a linear double-stranded DNA genome (160–175 kb), encapsidated in an icosahedral protein capsid that is surrounded by a tegument layer and an outer lipid bilayer envelope containing glycoproteins (reviewed in [8]). Upon infection, viral DNA is delivered to the nucleus, where it circularizes to a functional circular minichromosome and persists as a non-integrated episome for the lifetime of the host (reviewed in [9]).

Inside the host cell, KSHV exhibits a biphasic life cycle consisting of a life-long reversible latent phase and a transient lytic reactivation phase, which are distinguished by their virtually distinct gene expression profiles [10]. During latent infection, KSHV genome persists as a circular episome in the infected cell with a restricted latent gene expression without the production of progeny virions. The limited region within the KSHV genome, which is transcriptionally active during latency, encodes for four major open reading frames (ORFs), consisting of ORF73/Latency-associated nuclear antigen (LANA), ORF72/viral-cyclin (v-Cyc), ORF71/viral FLICE-inhibitory protein (v*-*FLIP), and ORFK12/Kaposins, along with 18 mature miRNAs (at last count) and viral interferon regulatory factor-3 (vIRF3) (reviewed in [11]). KSHV has a propensity to cause latent infection that is tightly regulated by the host immune responses and has been reported to play a significant role in the development of KSHV-associated malignancies. Since the KSHV genome does not encode any viral components required for latent DNA replication, Latency Associated Nuclear Antigen (LANA), the multifarious latent protein, is considered necessary (and sufficient) for latent viral episomal DNA replication and segregation, ensuring equal distribution of replicated episomes to each daughter cell during mitosis. To achieve this, LANA binds to the LANA-binding sequences within the terminal repeat (TR) region of the KSHV genome and tethers it to the host mitotic chromosomes via interaction, with several cellular chromatin-binding proteins, followed by replication of the viral genome using a *cis*-acting sequence in the TR region as a replication origin [12].

Global analysis of the viral gene expression of KS tumor cells indicated that KSHV predominantly expresses viral latent transcripts with only a few percent of cells being lytically active at any specific given time [13]. The latent phase of the viral life cycle is reversible and can be reactivated to enter into the second, well-ordered program of viral gene expression,* i.e.*, lytic reactivation. This phase predominantly consists of: (1) *KSHV Reactivation from latency*, followed by (2) *Lytic DNA Replication* and virion production. Upon reactivation from latency, a full repertoire of lytic viral genes are activated in a temporally regulated manner, leading to the transcriptional activation of three classes of lytic genes, namely, immediate early (IE), early (E), and late (L) genes [14,15,16]. The cellular machinery is switched on for an extensive viral DNA replication and gene expression, resulting in the assembly and release of infectious mature virion particles that egress out of the cell on disruption of the host-cell membrane. KSHV reactivation and lytic replication are not only important for viral propagation but also critical for KSHV-induced tumorigenesis.

Members of all three classes of lytic viral genes encode for proteins that assist in the formation of infectious virions [17]. The IE-lytic genes primarily govern the transition of KSHV genome from latent-to-lytic phase and consist of ORF50/RTA, ORF45, K8α, K8.2, K4.2, K4.1, K4, ORF48, ORF29b, K3, and ORF70. These genes are expressed within 10 h of induction and encode viral proteins that are directly involved in gene transcription and cellular modifications for viral replication. A series of studies have established that a single major IE-lytic protein-RTA acts as the quintessential latent-lytic switch that redirects KSHV to enter the productive transcriptional program required for viral spread and KS tumorigenesis. RTA protein (691 aa and 110 kDa) is the only viral lytic protein, both necessary and sufficient to disrupt latency and promote complete lytic cascade [18]. The RTA gene is reported to auto-activate its own promoter and transactivate the expression of multiple downstream lytic genes, including K8, K5, K2, K12, ORF6, ORF57, ORF74, K9, ORF59, K3, ORF37, K1, K8.1A, ORF21, vIL-6, PAN RNA, vIRF1, K1, and ORF65, either by itself (through RTA-responsive element, RRE) or in accord with other viral regulatory genes [19]. These E-lytic genes are expressed between 10–24 h post-induction and encode viral proteins primarily required for DNA replication and gene expression. The l-lytic genes that appear after 48 h post infection consist of viral structural proteins, including membrane glycoproteins (gB and K8.1), and a small viral capsid antigen required for assembly and maturation of the virions [20].

RTA plays an important role as both an initiator and a controller of KSHV lytic DNA replication [21]. Unlike latent DNA replication, lytic DNA replication: (1) depends on KSHV-encoded replication proteins; (2) initiates from a different origin (ori-Lyt); (3) replicates via a rolling-circle mechanism; and (4) leads to a multifold amplification of the viral DNA. The lytic origin of replication (ori-Lyt) consists of a specific origin binding protein (OBP) that plays a significant role in recruiting the core replication machinery to the site of replication. The two ori-Lyt domains, namely left ori-Lyt (ori-Lyt-L) and right ori-Lyt (ori-Lyt-R), are located between K4.2 and K5 and between ORF69 and ORF71, respectively, in the KSHV genome [22,23]. The ori-Lyts contain regions for various transcription factor-binding sites and RRE element that is essential for RTA-binding and ori-Lyt dependent DNA replication [22,23]. 

Despite the induction of lytic cycle following KSHV infection, there is a rapid inhibition of RTA promoter that further decelerates the full-blown KSHV reactivation [24]. The mechanisms that regulate the temporally ordered activation and genome-wide repression of lytic genes during primary infection are beginning to be resolved [25]. As both phases of KSHV life cycle are important for the development of KS and associated disorders, further understanding of the underlying mechanisms that coordinate regulation of gene expression may advance our knowledge of KSHV virology and assist in designing preventive therapeutic agents against KSHV lytic replication and associated tumorigenesis.

KSHV reactivation is an extremely complex process that involves a combination of both viral and cellular factors including but not limited to, temporary or permanent immune suppression, oxidative stress, inflammatory cytokines, hypoxia, viral co-infection and treatment with chromatin modifying agents. Thus far, a number of factors have been reported to stimulate or inhibit major viral proteins, however, the physiological relevance of these stimuli or repressors is far from being fully elucidated. In the following sections of this review, we will summarize recent studies that highlight the activation of KSHV lytic cycle and replication and will primarily focus on the relevant physiological, environmental, cellular, and viral regulatory factors involved in the regulation of KSHV’s biphasic life cycle, gene expression, and viral infection.

## 2. LANA and KSHV Reactivation

The two major KSHV proteins-LANA and RTA are shown to interact with each other and control the switch between latency and lytic reactivation [26]. Studies from multiple research groups reported a tremendous increase in the expression of several IE-lytic genes including RTA, MTA, vIL-6, ORF59, and K8.1 in 293T cells following deletion of LANA, indicating LANA-associated repression of basal level of RTA promoter as well as other RTA-responsive promoters [27,28]. LANA is shown to interact with RTA promoter and inhibit RTA gene expression via functional interaction with a recombination signal binding protein for immunoglobulin κ J region (RBP-Jκ protein), which is a major transcriptional repressor of the Notch signaling pathway [29]. LANA-mediated repression of RTA promoter and RTA auto-activation depends on RBP-Jκ binding sites. LANA recruits RBP-Jκ protein to repress the expression of RTA gene and down-regulates RTA self-activation by competing with RTA in RBP-Jκ-binding. In addition, RTA protein itself contributes to the establishment of KSHV latency by activating LANA protein expression following *de novo* infection. Therefore, the molecular transition between latency and lytic reactivation is controlled by the interplay between LANA and RTA proteins in KSHV-infected cells.

DNA methylation or CpG dinucleotide methylation, associated with the transcriptional silencing, also plays a key role in the induction of KSHV lytic cycle as the treatment of PEL-derived cell lines with DNA methyltransferase inhibitor, 12-o-tetradecanoylphorbol-13-acetate (TPA) or 5-Azacytidine (5-AzaC) caused demethylation of lytic promoters and induced KSHV lytic phase* in vitro* [30]. Bisulfite sequencing of latently infected BCBL-1 cell lines revealed hypermethylation of functionally conserved RTA gene of KSHV genome by *de novo* methyltransferases DNMT3a/DNMT3b and establishment of methylation marks exclusively on RTA promoter, leading to gene silencing during latency [30,31,32]. Recent studies by Grundhoff’s group reported a comprehensive tempo-spatial analysis of DNA methylation in several tumor-derived cell lines, as well as *de novo* infected endothelial cells using high resolution tiling microarrays together with immunoprecipitation of methylated DNA (MeDIP) [32]. These studies revealed that the KSHV genome is indeed subjected to hypermethylation at CpG dinucleotides, leading to the distinct, genome-wide DNA methylation patterns that include extensive methylation of lytic promoters followed by a poised state of repression during latency.

Interestingly, post-translational modifications of LANA, such as arginine methylation, phosphorylation and SUMOylation, have been shown to down-regulate the expression of lytic genes during the establishment of latency [28,33,34,35]. Treatment of BCBL-1 cells with histone deacetylase inhibitors, including sodium butyrate (NaB) and trichostatin A (TSA), caused a rapid dissociation of LANA from the RTA promoter and initiated transcription activation of RTA gene [28]. Furthermore, reports on phosphorylation of LANA by several kinases including glycogen synthase kinase (GSK-3β), DNA-PK/Ku and Pim-family kinase members, Pim-1 and Pim-3, have been reported to promote viral reactivation by negative modulation of LANA function [36,37,38]. LANA is also identified as a substrate for protein arginine methyltransferase 1 (PRMT1) and methylation at R20 site is found to influence strong binding of LANA to the KSHV genome and repression of lytic genes [39]. LANA is proposed to enhance histones (H2A and H2B) SUMOylation on the local chromatin by recruiting SUMO-Ubc9 complexes through SUMO-binding, resulting in a condensed chromatin and silencing of the KSHV genome (reviewed in [40]).

The early stage of KSHV infection is defined by the constitutive expression of latent genes, as well as temporally ordered expression of viral lytic genes. Recent genome-wide ChIP-seq studies described the epigenetic map of KSHV episomes during latency and indicated that chromatin of the KSHV genome is enriched with both active (H3ac or H3K4me3) and repressive histone marks (H3K9me3 and H3K27me3) [32,41]. Based on studies reported by several independent groups, KSHV-encoded latent genes are found to be associated with activating H3ac/H3K4me3-histone marks, whereas KSHV-encoded IE and E-lytic genes are found to possess either a H3ac/H3K4me3-rich euchromatin or a H3ac/H3K4me3 and H3K27me3-rich bivalent chromatin, and L-lytic genes are found to have increased levels of heterochromatin-associated repressive H3K9me3 and H3K27me3-histone marks. In addition, H3K9 histone demethylase JMJD2A, and H3K27 histone methyltransferase EZH2 of the Polycomb Repressive Complex 2 (PRC2), predominantly bind to the KSHV genome and their recruitment by LANA is shown to maintain H3K27me3-associated silencing marks on lytic genes and repress their expression during latency (reviewed in [9]). Decrease of H3K27me3 marks, by either transient expression of UTX/JMJD3, or by blocking with EZH2 of PRC2 complex, disrupts latency and induces lytic reactivation [32,41]. As LANA is continuously expressed following *de novo* infection/during latency and interacts with several transcriptional repressors (heterochromatin protein HP1α, methyl-CpG-binding protein MeCP2, histone deacetylase co-repressor mSin3 and DNA methyltransferases) and chromatin-remodeling proteins (H3K9me3 histone methyltransferase SUV39H1 and hSET1 complexes, H3K9 demethylase KDM3A, histone acetyltransferase CBP, histone deacetylase mSin3 and chromatin transcription complex FACT), it is evident that LANA helps to silence lytic gene expression and promotes KSHV latency through epigenetic control [42,43,44,45,46,47].

## 3. Stimulus Triggering KSHV Reactivation

Thus far, several PEL-infected cells, endothelial cells, CV-1, human fibroblasts and HEK cells are known to maintain KSHV in the latent form that can be induced to enter the complete productive cycle of KSHV, following treatment of cells with the broad-spectrum protein kinase C-activator (TPA) or histone deacetylase inhibitor (NaB) (reviewed in [48]). As a result, these cell lines serve as an authentic tumor model to study KSHV life cycle, providing several insights into the numerous cellular pathways that control viral reactivation. As these chemicals target numerous cellular and viral pathways, it appears that more than one mechanism is necessary to reactivate KSHV. More recently, KSHV was found to efficiently infect, immortalize, and transform, rat embryonic metanephric mesenchymal precursor (MM) cells [49]. KSHV-transformed MM cells (KMM) support the growth of KSHV-induced tumors, hence, providing a novel animal model to study the intrinsic oncogenic pathways underlying KSHV latency and reactivation.

### 3.1. Viral Co-Infection

While KSHV infection appears to be necessary for the development of KS, the immunodeficiency appears to be another significant factor, as the immunosuppressed patients are often susceptible to many other infectious agents [50]. Several viral proteins, including HIV-1 trans-activating protein (HIV-1 tat) [51], HIV-1 negative factor protein (HIV-1 Nef) [52], herpes simplex virus type 1 (HSV-1) [53], herpes simplex virus type 2 (HSV-2) [54], human cytomegalovirus (HCMV) [50], human herpesvirus-6 (HHV-6), herpes simplex virus type 2 (HSV-2) [54], human cytomegalovirus (HCMV) [50], human herpesvirus-6 (HHV-6) [55], human herpesvirus-7 (HHV-7) [56], and papillomavirus [57] are proven to be potent cofactors that can activate KSHV lytic replication and influence KSHV pathogenesis. In addition, it has been demonstrated that inflammatory cytokines, such as oncostatin M (OSM), hepatocyte growth factor (HGF), interferon-γ (IFN-γ) [58], and toll-like receptors 7 and 8 (TLR7/8), when stimulated by viral infections, can trigger KSHV reactivation (reviewed in [59]).

### 3.2. Hypoxia

As an important co-factor, hypoxia (low tissue oxygen concentration) is physiologically linked with the initiation and progression of KSHV-associated cancers and known to induce the accumulation of hypoxia-inducible factors (HIF-1α/2α) (reviewed in [59]). Hypoxic stress in PEL cells is shown to stimulate KSHV lytic reactivation through accumulation of HIF-1α within the hypoxia-responsive elements (HRE, 5'-RCGTCG-3') region of RTA promoter, and accumulation of HIF-1α/2α within the HRE2 regions of ORF34-37 promoters. Hypoxia also triggers the activation of plasma cell-differentiation factor X-box binding protein 1 (XBP-1) that trans-activates the KSHV RTA promoter with HIF-1α, leading to the expression of RTA protein and reactivation from latency [60]. Splicing of XBP-1 mRNA, an event that occurs during B-cell differentiation, is also critical for disrupting latency and promoting KSHV reactivation, with the possibility of integration of latter into the host cell differentiation program. In addition, under hypoxic conditions, LANA is reported to interact with HIF-1α bound to HREs within the RTA promoter to upregulate its gene expression [61]. Recent studies by Cai *et al.* have demonstrated that LANA interacts with a new host nuclear protein and hypoxia-sensitive chromatin remodeler, KAP1 (KRAB-associated protein 1), through its SUMO-2 interacting motif (LANA^SIM^), and recruits it to the lytic promoter region of the KSHV genome for transcriptional repression [62]. Inhibition of KAP1 in KSHV-infected PEL cells enhanced the hypoxia-induced lytic reactivation through association of RBP-Jκ with HIF-1α within the RTA promoter region [63]. In KSHV-harboring cells, shRNA knockdown of KAP-1 resulted in the induction of lytic genes and a five-fold increase of RTA-mediated lytic reactivation.

### 3.3. Oxidative Stress and Reactive Oxygen Species (ROS)

As all forms of KS are characterized by increased levels of inflammation and oxidative stress, it is postulated that reactive oxygen species (ROS), such as hydrogen peroxide (H_2_O_2_), mediate KSHV reactivation from latency (reviewed in [59]). A recent report showed that hypoxia and pro-inflammatory cytokines-mediated spontaneous KSHV reactivation and lytic replication are supported by hydrogen peroxide (H_2_O_2_) through both autocrine and paracrine signaling [64]. H_2_O_2_ is sufficient for inducing and mediating KSHV lytic replication in KS tumors by activating ERK1/2, JNK, and p38 mitogen-activated protein kinase (p38 MAPK) pathways [65]. Significantly, treatment with antioxidant/H_2_O_2_ scavengers; *N*-acetyl-l-cysteine (NAC), catalase and glutathione peroxidase inhibits KSHV lytic replication and tumor progression* in vivo* and slows down the development of KSHV-induced lymphoma in a mouse xenograft model [64]. Another study reported that, in infected PEL cell lines BC-3 and BCBL-1, increased levels of reactive oxygen species (ROS) may induce oxidative stress that can trigger transcriptional activation of KSHV lytic cycle and promote cell death [66]. Additionally, ROS levels can be upregulated by NF-kB inhibition and treatment of infected cells with an increased amount of NF-kB inhibitor than used for inducing KSHV reactivation,,can further elevate ROS levels and induce apoptosis [66]. In addition, p38 signaling and anti-cancer drugs (cisplatin and arsenic trioxide) are found to induce KSHV lytic cycle and host cell death in an ROS-dependent manner [66]. These results directly relate KSHV reactivation to oxidative stress and inflammation, suggesting that the antioxidants and anti-inflammation drugs could be potential drugs for effectively targeting KSHV lytic replication and KSHV-associated tumorigenesis.

### 3.4. Histone Deacetylases and Histone Deacetylase Inhibitors (HDACs and HDACi)

**Figure 1:**
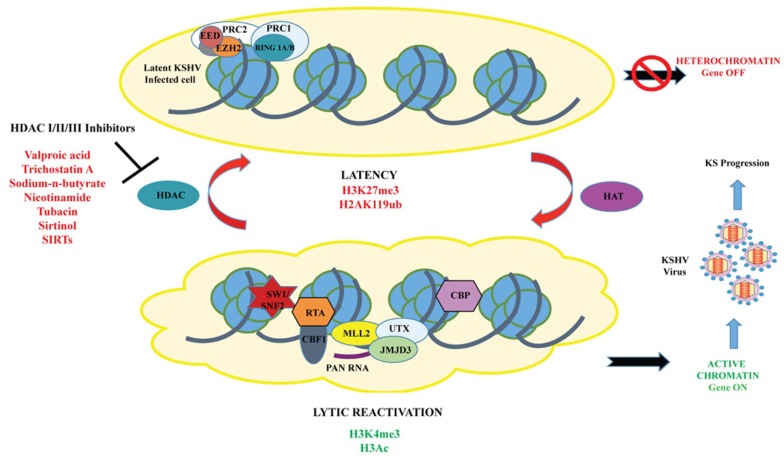
A model for the chromatin landscape of RTA promoter during KSHV latency and lytic reactivation. During latency, the chromatin of RTA promoter is enriched in both activating (H3ac/H3K4me3) and repressive (H3K27me3)-histone marks, as well as the transcription repressors (Polycomb Repressive Complex 2 and HDACs), hence, the RTA promoter is transcriptionally silent. Following reactivation, the bivalent chromatin of RTA promoter is remodeled into transcriptionally active euchromatin by histone modifying enzymes, such as histone acetylases (HAT/CBP), H3K27me3 demethylase (UTX/JMJD3), H3K4 methyltransferase (MLL complex), and inhibitors of HDACs (Valproic acid, trichostatin A, NaB, nicotinamide, sirtinol, tubacin, and SIRTs, leading to the production of infectious KSHV virious and progression of KSHV-induced malignancies.

Several research groups have reported that HDAC Class I, II, and III can regulate KSHV reactivation, and activation of lytic gene expression can be triggered by treatment of KSHV latent cells with HDAC inhibitors [9,67,68]. HDACs are a group of enzymes that remove acetyl groups from ε-*N*-acetyl lysine amino acids in histones/proteins and play an important role in the regulation of gene expression. As mentioned earlier, during latency, IE and E-lytic genes possess bivalent chromatin associated with both repressive (H3K9me3 and H3K27me3) and activating (H3K4me3, H3ac and H3K9/K14-ac)-histone marks. In addition, previous studies showed that demethylation of H3K27me3 using UTX or dissociation of the histone methyltransferase EZH2, counteracts PRC2 repression of the RTA promoter [32,41]. In order to determine which HDAC classes (Class I and II) regulate KSHV latency and reactivation, five latently infected Vero- and PEL-cell lines were treated with a series of HDACi, including Valproic acid (VPA), trichostatin A (TSA), nicotinamide, sirtinol, tubacin, and NaB (Figure 1) [67]. The results indicated that HDAC class I inhibitors of were sufficient enough to induce KSHV virus and lytic gene expression with varied reactivation potential. Out of all the HDACi tested, VPA was found to be the most effective inducer of lytic cycle gene expression, followed by TSA. The data suggested that inhibition of HDAC class I molecules, alone, is sufficient to reactivate KSHV but the inhibition of class I and IIa molecules, together, is optimal for reactivation [67].

Additionally, Gao’s research group recently determined the role of Class III HDACs inhibitors or sirtuins (SIRTs) on the KSHV life cycle and reactivation by treatment of KSHV-positive PEL cell lines (BCP-1, BC-3 and BCBL-1) with three distinct HDACi, namely-nicotinamide (NAM), sirtinol and NaB [68]. The studies revealed that both NAM and sirtinol could efficiently reactivate KSHV from latency. In addition, it was shown that SIRT1 is involved in the control of latency and can prevent the expression of several downstream genes due to its interaction with the RTA promoter [68].

### 3.5. Dietary Supplements

A recent report determined that Resveratrol (Rev), an important dietary supplement, inhibits KSHV reactivation by altering the interactions between early growth response-1 (Egr-1) and the RTA promoter [69]. Electrophoretic mobility shift assays (EMSA) and chromatin immunoprecipitation (ChIP) experiments revealed that Egr-1, a cellular transcription factor known to play a critical role in the replication of several viruses, may potentially bind to the KSHV RTA promoter via at least two different GC-rich binding regions and follow a similar expression profile during *de novo* KSHV infection. Elevated cellular Egr-1 expression is reported to enhance viral RTA expression in a Raf > MEK > ERK-dependent manner [69]. Further, Rev is found to lower ERK1/2 activity and expression of Egr-1 in KSHV-infected cells, resulting in the suppression of virus reactivation from latency, though the precise mechanism by which Rev regulates KSHV reactivation is still unclear [69].

## 4. Role of Viral and Cellular Proteins Important for Lytic DNA Replication

KSHV lifecycle undergoes a transition between a dormant, latent phase and an active lytic replication phase [15,59]. KSHV lytic DNA replication requires the expression of at least eight viral genes including: ORF9 (DNA polymerase), ORF6 (single-stranded DNA binding protein), ORF40/41 (primase-associated factor), ORF44 (helicase), ORF56 (primase), ORF59 (processivity factor), ORF50 (replication and transcription activator or RTA), and ORF K8 (K-bZIP) [70,71]. RTA, an immediate early protein, is the most important protein required for the activation of lytic replication, transcription initiation, as well as recruitment of additional factors (reviewed in [48,59,72]). This section will describe several viral, as well as cellular, proteins that are important for lytic reactivation. 

### 4.1. Viral Factors

#### 4.1.1. K-RTA (KSHV Replication and Transcription Activator)

KSHV encoded ORF50/ RTA (replication and transcription activator), is a key regulator for the lytic reactivation from viral latency [15,73]. Expression of RTA is both essential and sufficient for KSHV reactivation [71,73,74]. Genetic mutation of RTA results in impaired reactivation and lytic DNA replication [75]. RTA has been reported to be phosphorylated [76,77], Poly (ADP-ribosyl)ated [77] and ubiquitinated [78]. RTA also autoactivates its own promoter [19] and transactivates other important lytic genes, including vIL-6 [79,80] polyadenylated nuclear RNA (PAN) [81] ORF57 (MTA) [82], ORF59 (PF8) [83,84], K-bZIP [82], vIRF1 (ORF-K9) [85], ORF-K1 [86], small viral capsid protein (ORF65) [87], ORF56 [88], SOX (ORF37) [89], vOX [90], and ORF52 [79]. RTA binds and transactivate many promoters containing K-RTA response element (RRE) [91]. KSHV LANA is also known to repress lytic reactivation, as well as RTA-mediated autoactivation [92]. LANA-mediated suppression of RTA autoactivation is dependent on RBP-J*κ*, which competes with RTA for binding to RBP-Jκ [29]. Lytic reactivation results in the acetylation of LANA, leading to the dissociation of LANA from the ORF50 promoter bound to Sp1 [28]. Genome-wide screening revealed a consensus RTA interaction motif, TTCCAGGAT(N)(0–16)TTCCTGGGA [93,94]. In addition, specific amplification of bound sequences* in vitro* showed a number of RTA direct binding targets [93], such as ORF8, ORFK4.1, ORFK5, PAN, ORF16, ORF29, ORF45, RTA, K-bZIP, ORFK10.1, ORF59, ORFK12, ORF71/72, vOX/vGPCR (ORF74), ORF-K15, the two oriLyts, and the miR cluster [94]. These variations indicate that RTA cooperatively binds to its targets by associating with other regulatory proteins [79,93]. Additionally, RTA activates its own promoter by binding to the Oct-1 transcription factor and RBP-Jκ [19,95]. Furthermore, RTA mediated transactivation of viral lytic promoters, such as MTA and thymidine kinase (TK, ORF21), depends on Sp1, octamer-binding protein-1 (Oct-1), and XBP-1 [96,97,98]. However, direct binding of RTA to its promoter is not critical for its autoactivation [95,99]. Several other recent studies have also shown that RTA is recruited to RREs through interaction with RBP-Jκ [100,101]. Similarly, in a recent study using recombinant viruses with deleted RBP-Jκ sites within RTA promoter showed an increased viral latency and a reduced efficiency for lytic replication [102]. In addition, RTA stimulates the Notch signaling pathway, RTA mediated intracellular-activation of Notch1 is sufficient to reactivate KSHV from latency to the lytic replication cycle [103,104].

RTA transactivation of viral promoters also depends on its interactions with other cellular proteins. RTA recruits CREB binding protein (CBP), the SWI/SNF chromatin-remodeling complex, and the TRAP/mediator coactivator into viral promoters [105]. RTA binding positively regulates Histone acetyl transferase (HAT) activity of CREB [106]. A recent report showed that RTA transactivates cellular Bcl-2 through targeting of CCN_9_GG-like RTA responsive elements (RREs) for lytic reactivation and enhanced virion production [107]. Furthermore, it has been shown that K-RTA associates with a homologue of the Kruppel-associated box-zinc finger proteins (KRAB-ZFPs), for its transactivation function [108,109,110]. The co-repressor of K-RBP, Kruppel-associated box domain-associated protein-1 (KAP-1), is a cellular transcriptional repressor that regulates chromosomal remodeling, participates in the maintenance of latency by repressing lytic promoters [111]. During latency KAP-1 binds to viral lytic promoters to repress gene expression and depletion of KAP-1 is sufficient to induce KSHV reactivation [62]. Studies show that sumoylation and phosphorylation are required to regulate KAP-1 association with heterochromatin [62,111]. KAP-1 is phosphorylated at Ser 824, during lytic reactivation, resulting in decreased sumoylation and association to the condensed chromatin on viral promoters [111]. A recent study confirmed that KAP1 is targeted by KSHV-encoded latency-associated nuclear antigen (LANA) to repress the transactivation of K-RTA [112]. Additionally, knockdown of KAP1 in KSHV-infected primary effusion lymphoma (PEL) cells reduced viral episome stability and enhanced the efficiency of KSHV lytic reactivation by hypoxia, suggesting that both KAP1 and the cooperative interaction of RBS HRE within the RTA promoter are crucial for KSHV latency and hypoxia-induced lytic reactivation [63]. K-RTA interacts with K-bZIP, and increasing evidence indicates that repression of K-RTA transactivation by K-bZIP, a basic leucine zipper (bZIP) transcription factor encoded by KSHV, is essential for the modulation of lytic DNA replication by a feedback circuit [70,113,114]. RTA also interacts with C/EBPα, and the cooperative interaction of K-bZIP and RTA with C/EBPα is essential for the activation of K-bZIP promoter by binding to a proximal C/EBP*α* binding site [115]. The promoters of RTA, PAN, and MTA are activated through direct interaction of the C/EBPα and RTA complex [116]. K-RTA is also shown to be associated with viral ORF59, a processivity factor for viral DNA polymerase, and ORF45, a multifunctional tegument protein required for lytic replication [117,118].

Recent studies showed that K-RTA activity is regulated by its association with cellular peptidyl-prolyl cis/trans isomerases (PPIase), Pin1. Pin1 binds specifically to phosphorserine or phosphorthreonine-proline (pS/T-P) motifs in the K-RTA and enhances K-RTA transactivation [119]. Additionally, it has been shown that K-RTA is regulated by a 48aa small peptide, vSP-1, encoded by a polyadenylated RNA of 3.0 kb (T3.0), transcribed from the opposite strand of the KSHV RTA (ORF50) DNA template. vSP-1 associates with RTA at the protein abundance regulatory signal (PARS) motifs, and this interaction prevents RTA from degradation by ubiquitin-proteasome pathways, thus, facilitating KSHV lytic replication [120]. Apart from direct DNA interaction, RTA also cooperates with various host transcriptional factors to transactivate several downstream viral genes. Additionally, K-RTA exhibits an ubiquitin E3 ligase activity, RTA is auto-ubiquitinated and directs several cellular and viral proteins for proteasome-mediated degradation [121]. One of the cellular proteins targeted by RTA is Hey1, which interacts with repressor mSin3A. This, in turns, downregulates the expression of RTA by direct interaction with the RTA promoter [122]. RTA upregulates its own expression through ubiquitin-mediated targeting of Hey1 for degradation. Another cellular protein targeted by RTA mediated degradation is IRF-7, a critical modulator of type I IFN induction [78]. IFN signaling plays a crucial role in repressing KSHV lytic replication, therefore, this finding indicates that RTA might circumvent these cellular innate immune defenses during lytic reactivation. Direct association of RTA to the origin of lytic DNA replication (oriLyt) has been demonstrated [21]; there are two distinct oriLyt regions in the KSHV genome [23]. The left oriLyt (oriLyt-L) lies between ORFK4.2 and K5 and is comprised of a region encoding numerous transcription factor binding sites, an A+T-rich region, and a G+C repeat. Similarly, the right oriLyt (oriLyt-R) is situated between ORF69 and vFLIP and is an inverted duplication of oriLyt-L. Importantly, both oriLyts contain RREs, and [123] a direct interaction of RTA to RREs is critical for oriLyt-dependent DNA replication [23,70,123]. The presence of RREs and a downstream TATA box indicate that this region may serve as an RTA-dependent promoter, and a transcription event may be required for oriLyt-dependent DNA replication [123]. Additionally, recent studies have identified that K-RTA is able to function as a STUbL, which is capable of ubiquitylation of SUMO and SUMO conjugates* in vitro* and* in vivo*. Thus, K-RTA is an ubiquitin ligase, preferentially targeting SUMO-containing proteins for ubiquitylation; including sumoylated K-bZIP and promyelocytic leukemia (PML) nuclear bodies [124]. Together, these results suggest that RTA is a master regulator of viral lytic DNA replication. 

#### 4.1.2. ORF57-mRNA Transcript Accumulation (MTA)

ORF57 is a viral early protein, which favors viral intron-less transcript accumulation, transports, and enhances splicing of intron-containing viral RNA transcripts [125]. MTA is essential for KSHV lytic replication, moreover, genetic knockout of MTA disrupts KSHV productive lytic replication [125,126]. MTA protein carries domains with putative transcriptional and post-transcriptional functions [127]. MTA directly associate with RTA and both proteins are detected in the RTA promoter during lytic replication. KSHV MTA associates with DNA, which was identified by gel shift and chromatin immunoprecipitation assays [127,128]. In addition, it has been shown that MTA directly associates with K-bZIP protein and binds to promoter as well as transcribed regions of PAN RNA, K4, and K-bZIP [129]. These reports suggest that MTA stimulates RNA export through its association with Aly/REF, a cellular RNA-binding protein acting as an adaptor for the nuclear RNA export receptor NXF1/TAP [130]. Additionally, recent studies suggest that Aly/REF-ORF57 association does not necessarily play any significant role in the ORF57-mediated enhancement of ORF59 expression, as Aly/RE knockdown in host cells did not affect the function of ORF57 [131,132]. MTA enhances the expression of RTA or other lytic genes, most probably by binding to transcription regulatory proteins. Further, MTA cooperates with RTA to modulate the viral gene expression in a cell-line-specific manner [127,128]. It is suggested that a putative A/T hook domain within MTA arbitrates DNA binding and transcriptional initiation [127].

MTA modulates a cascade of viral gene expression and accumulation of specific viral and cellular mRNAs during lytic replication [132]. Physical association of MTA and RTA is essential for the synergistic regulatory effect of MTA. When RTA’s transactivation function is removed, MTA no longer affects the expression of viral genes, indicating that their cooperative effect depends on RTA’s transactivation function [128]. It has been shown that MTA regulates mRNA accumulation. Further, a recent study employing a genome-wide CLIP (cross-linking and immunoprecipitation) approach detected KSHV PAN, a long non-coding polyadenylated nuclear RNA, as an important target of ORF57 [133]. Genetic disruption of ORF57 affects PAN RNA expression. In co-transfection experiments, expression of exogenous ORF57 alone increased PAN RNA expression by 20–30-fold, which was due to the MRE (MTA responsive element) at the 5' PAN RNA, however, not as much on an ENE (expression and nuclear retention element) at the 3' end of PAN RNA. Further studies showed that the major function of the 5' PAN MRE is to increase the half-life of PAN in the presence of ORF57 [133]. Systematic mutational analyses identified a core motif, consisting of nine nucleotides, in MRE-II, which is essential for ORF57 interaction and function. The 9-nt core in MRE-II also interacts with cellular poly (A)-binding protein C1 (PABPC1) [134], but not E1B-AP5, which binds to another region of MRE-II [133]. In the presence of ORF57, PAN RNA is partially exportable, suggesting that ORF57 functions to accumulate a non-coding viral RNA during the course of lytic infection [133,134]. Additionally, MTA is also shown to stabilize RNAs and activates translation of mRNAs that carry internal ribosome entry sites [135]. It has been also shown that KSHV ORF57 specifically binds to ORF59 RNA and associates with cellular RNA export cofactors RBM15 and OTT3 to enhance the expression of ORF59 [136].

#### 4.1.3. KSHV K8-K-bZIP—Lytic Replication-Associated Protein (RAP)

K-bZIP is a basic leucine zipper-containing protein encoded by KSHV K8 [137]. The K-bZIP gene locus consists of two promoters: one early promoter controlling K-bZIP and the second late promoter controlling K8.1 [91,138]. K-bZIP directly binds to K-RTA through K-bZIP’s basic domain and a specific RTA region [139,140]. Further, association of K-bZIP suppresses K-RTA transactivation of the MTA promoter in a dose-dependent manner [109,110]. Recent studies suggest that K-bZIP is not required for lytic reactivation in KSHV BACmid systems [114,141,142], however, it was reported to be crucial for virus production in infected PEL cells [143]. It has been shown that K-bZIP interacts with oriLyt [22,144,145,146] and is critical for oriLyt-dependent DNA replication in a plasmid-based transient expression system [70], but its absence can be complemented by an over-expression of RTA [145]. Similarly, association of cellular transcription factor CCAAT/enhancer-binding protein α (C/EBPα) to K-bZIP has also been shown to increase the expression and stabilization of C/EBPα and p21CIP1 proteins, followed by G0/G1 cell cycle arrest [18,115]. Similarly, KSHV bZIP can also bind to the positive regulatory domain I/III region of the IFNb promoter to block IRF3-mediated IFNb transcription [147,148]. In addition, K-bZIP represses the RTA autoactivation [139] and colocalizes with HDAC1/2 through the leucine zipper domain without the requirement of sumoylation of K-bZIP [149]. K-bZIP is phosphorylated on residues Thr111 and Ser167 by a serine/threonine protein kinase (vPK) encoded by ORF36 [146,150]. However, phosphorylation at T111 has a negative effect on both the extent of sumoylation and the repressive activity of K-bZIP [150]. K-bZIP is sumoylated at residue lysine 158, and this sumoylation is essential for K-bZIP mediated transcription repression [146]. As a SUMO adaptor, KbZIP represses transcription by recruiting Ubc9 to specific viral promoters [146]. In addition, it has been shown that K-bZIP functions as the viral SIM-containing poly-SUMO-specific E3 ligase, with specificity for SUMO-2/3 [35]. Further, K-bZIP catalyzes its auto-sumoylation and the sumoylation of other K-bZIP-interacting proteins, such as p53 and pRB [148]. 

A genome-wide analysis of K-bZIP’s transcriptional regulation on KSHV gene promoters showed that RTA activated 34 viral promoters whereas K-bZIP alone activated 21 promoters [140]. Nonetheless, when RTA and K-bZIP were combined together, K-bZIP was found to repress three RTA-responsive promoters, suggesting that K-bZIP might also transactivate some viral lytic genes during KSHV reactivation [140]. These data strongly suggest that K-bZIP plays a crucial during lytic gene expression and DNA replication in PEL cells [140,151]. Further, K-bZIP also directly binds to oriLyt, indicating that K-bZIP might be playing a crucial in lytic DNA replication [70]. Further, the interaction of K-bZIP with oriLyt is also modulated by LANA expression [145]. Taken together, these studies show that K-bZIP has dual independent functions in modulating the KSHV life cycle by facilitating lytic DNA replication or repressing the lytic gene expression as a feedback modulator [145]. Together with these results, knockdown of K-bZIP in latently infected BCBL-1 and BC-3 cells showed a significant reduction in the expression of RTA, MTA, and ORF26 transcripts, as well as decreased RTA and ORF-K8.1 protein levels, as well as defective viral DNA replication and virion production [143]. Collectively, these results suggest that K-bZIP regulates its own expression and possibly other RTA-transactivated lytic genes by a feedback loop.

#### 4.1.4. ORF59- Viral Processivity Factor

Kaposi’s sarcoma-associated herpesvirus (KSHV) ORF59 plays a critical role in viral lytic DNA replication as a DNA processivity factor to the viral DNA polymerase (ORF9) [70,83]. ORF59 is highly upregulated during lytic reactivation and *de novo* primary infection. ORF59 forms a homodimer in the cytoplasm and associates with ORF9 to translocate it to the nucleus during lytic DNA replication [152]. ORF59 associates with C/EBPα binding motifs within oriLyt and this binding is K-RTA dependent, where K-RTA acts as an initiator of lytic replication. Additionally, disruption of the K-RTA–ORF59 interaction by a dominant negative approach impairs oriLyt-dependent DNA replication [84]. This strongly suggests that the K-Rta-ORF59 interaction is crucial for lytic DNA replication. ORF59 is a phosphoprotein and is phosphorylated by KSHV viral Ser/Thr kinase, ORF36 primarily at Ser378, which is essential for ORF59's ability to bind to RTA and the oriLyt [83,84]. In a recent study, it has been shown that lytic infection of KSHV induces severe DNA double-strand breaks (DSBs) and impede non-homologous end joining (NHEJ) in host cells. Further, ORF59 was found to be associated with Ku70 and Ku86 and this association was dependent on DSBs, suggesting that KSHV lytic replication may induce tumorigenesis by causing DNA DSBs and interrupting the DSB repair of mechanism [153].

#### 4.1.5. ORF6-Single Strand Binding Protein

KSHV ORF6, a delayed-early gene encodes for a 126 kDa ssDNA binding protein that has been shown to participate in origin-dependent DNA replication [74,154,155]. The expression of ORF6 is regulated by RTA, which could bind to RBP-Jκ recognition site on the ORF6 promoter via interaction with the RBP-Jκ protein [95,155]. Genetic disruption analysis of the ORF6 gene, using the bacterial artificial chromosome (BAC) system, identified the functional role of ORF6 in lytic DNA replication. The mutant virus showed impaired DNA synthesis and failed to make progeny virions. Additionally, transient expression of ORF6 has rescued both defects, suggesting that ORF6 is critical for KSHV lytic replication [155]. 

### 4.2. Cellular Factors

**Figure 2:**
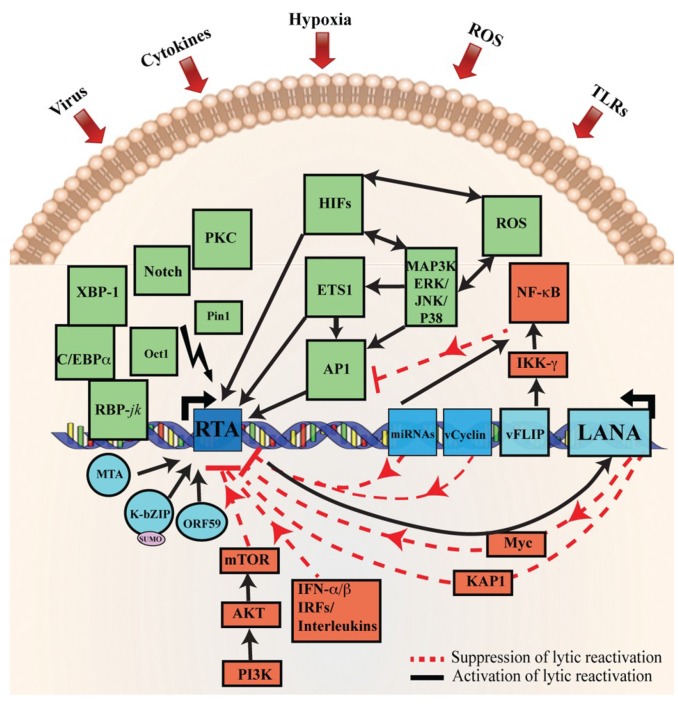
Schematic representation of cellular signaling pathways involved in KSHV latency and reactivation. During latency, KSHV latent genes, including LANA, vFLIP, miRNA, and vCyclin activate and maintain various cytokine-mediated cell proliferation and angiogenesis pathways, such as JAK/STAT, PI3K/AKT/mTOR, cMyc, and NF-κB, to suppress KSHV lytic reactivation. The red line represents the inhibitory pathways involved in the maintenance of KSHV latency. Disruption of these signaling pathways by various stimuli, such as secondary infection by bacteria, viruses, hypoxia, inflammatory cytokines, and oxidative stress upregulate RTA expression resulting in KSHV reactivation. The solid black arrows represent signaling pathways that are activated during KSHV lytic reactivation. Moreover, RTA, as well as RTA-induced KSHV genes MTA and K-bZIP, have been shown to interact with XBP-1 and C/EBPα to modulate various cellular signaling pathways. Deregulation of these cellular signaling pathways, such as MAPK, PKCd, b-Raf/MEK/ERK, PKA, Notch, RBP-Jκ, JNK, Pim-1/Pim-3, and TLR7/8 signaling by RTA lead to the reactivation of latently infected KSHV cells to lytic replication. This figure is adopted and modified from a previous review [59].

Several cellular signaling pathways are identified to be involved in the reactivation of KSHV from latency, such as PKCd [156], b-Raf/MEK/ERK [157], PKA [104], Notch and RBP-Jκ [95,158], p38 and JNK [159], Pim-1 and Pim-3 [160], PI3K and Akt [161], and TLR7/8 signaling [162]. Apart from these signaling pathways, a number of additional cellular factors also mediate KSHV reactivation [163,164,165,166,167] (Figure 2). It has been shown that intracellular calcium transport activates Ca++ dependent viral reactivation, and inhibition of calcineurin signaling, in turn, blocks KSHV reactivation [168]. Similarly, Protein kinase C delta (PKCdelta) plays a role in KSHV lytic replication [156]. Activation of the MEK/ERK, JNK, and p38 mitogen-activated protein kinase (MAPK) pathways play a central role during KSHV infection. Activation of the MAPK pathway, immediately after infection, enables the establishment of a successful KSHV infection [169,170]. Furthermore, MAPK pathways are induced during lytic reactivation [157,159,163]. Similarly, cellular MAP4K4 is also known to play a crucial role in inflammation, insulin resistance, and the invasiveness of several human malignancies [171,172]. Recently, it has been suggested that MAP4K4 act as a novel mediator of KSHV lytic reactivation from latency [172]. Similarly, yet another essential pathway mediating KSHV reactivation is the Raf/MEK/ERK/Ets-1 pathway [163]. Likewise, promoters of K-RTA, MTA, K-bZIP, and origins of lytic replication (oriLyt) have been shown to carry a functional DNA-binding site for AP-1 and are responsive to AP-1 activation [70,159,173]. During *de novo* infection, KSHV has been shown to induce MEK/ERK, JNK, and p38 MAPK pathways in human umbilical vascular endothelial cells (HUVEC). This, in turn, regulates AP-1 to facilitate its entry into the target cells and initiate a productive lytic replication at the early acute stage of infection [169,174]. Additionally, in latent KSHV-infected, cells these MAPK pathways modulate both spontaneous and TPA-induced KSHV reactivations and activate the expression of several transcription factors, such as AP-1 and Ets-1 [157,159,175].

KSHV lifecycle is also controlled by the viral protein, K-RTA by altering the Notch signaling pathway through binding with RBP-Jκ [95,101]. Additionally, Notch signaling and the expression of two Notch ligands (JAG1 and DLL4) are upregulated through KSHV genes, expressed during KSHV latent and lytic infection [176,177]. Similarly, Hypoxia-inducible factor (HIF) has been shown to induce numerous genes associated with angiogenesis and tumor growth, and the KSHV infected cells express elevated levels of HIF1α and HIF2α [177,178,179]. Furthermore, both LANA and vIRF3 have been shown to play roles in the stabilization of HIF1α via protein–protein interactions [61,178,180]. Secondary infections by other pathogens, such as HIV and bacteria, have been shown to trigger KSHV reactivation [162,181]. Similarly, short-chain fatty acids (SCFA) from periodontal pathogens suppress histone deacetylases HDAC1, EZH2, and SUV39H1 and downregulates the expression of silent information regulator-1 (SIRT1) to promote KSHV replication [182]. Cytokine-mediated JAK–STAT signaling also regulate various important biological processes, such as immune response, cell growth, and differentiation. KSHV infection has been shown to upregulate gp130 receptor expression, which leads to a constitutive phosphorylation of JAK2/STAT3 activation [183,184]. Further studies have revealed that both LANA and vGPCR play roles in the modulation of JAK2/STAT3 signaling to create angiogenic factors [185,186]. This is further confirmed by the LANA-mediated STAT6 phosphorylation through the inhibition of IL-4 for the maintenance of latency [187].

KSHV has evolved multiple mechanisms to manipulate cellular anti-apoptotic and survival pathways and disruption of these pathways reactivates KSHV [188,189,190]. Apart from AP-1, NF-κB also antagonizes RBP-Jκ to impair the expression and transactivation function of RTA [190]. Furthermore, inhibition of NF-κB pathway in latently infected cells disrupts viral latency and activates viral lytic replication [191]. However, the available data suggest that the role of the NF-κB pathway in the KSHV life cycle is context dependent [192]. It is very likely that the balance of AP-1 and NF-κB pathways decide the fate of virus replication status in a particular cell type [174,193,194]. Consistent with these findings, a recent study showed that inhibition of the pro-survival PI3K-Akt pathway favors KSHV reactivation from latency [195]. Furthermore, inhibition of the Akt pathway reactivates KSHV from latency by increasing the RTA expression [195]. KSHV encoded proteins are also known to modulate the cellular phosphatidyl inositol-3-kinase (PI3K)/AKT/mammalian target of the rapamycin (mTOR) signaling pathway to control cell proliferation. Cellular PI3K/AKT/mTOR signaling is a common to many growth factors and cytokine receptors [196]. However, thus far, only a few KSHV proteins have been shown to regulate PI3K/AKT/mTOR signaling, which include K1 [197,198], (vGPCR) [199,200], vIL-6 [183,201], and ORF45 [170,202]. 

## 5. Lytic Proteins in Controlling Immune Regulation and Pathogenesis

**Figure 3:**
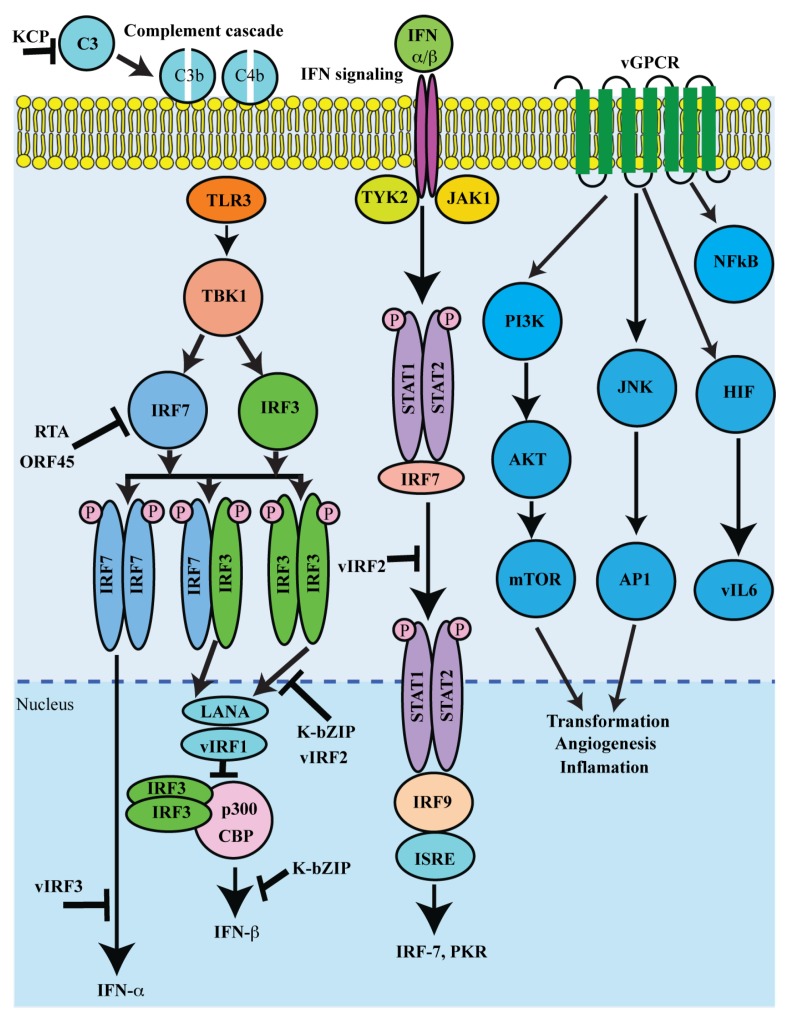
Schematic representation of lytic proteins in immune regulation and pathogenesis: The major immune functions targeted by viral lytic proteins include IFN production, interferon regulatory factor (IRF) activation, complement activation, inflammasome and chemokine activation. Regulating both IFN production and signaling is a potent antiviral defense, vIRF can bind and disrupt the transcriptional activities of IRF1, IRF3, and IRF7. Additionally, vGPCR is a constitutively active homologue of the IL8 receptor. vGPCR activates various cell signaling pathways and transcription factors to enhance the production of pro-inflammatory chemokines and cytokines, such as vIL-6. Furthermore, KSHV-encoded KCP regulates complement by increasing the decay of the classical C3 convertase.

Lytic reactivation results in an expression of several KSHV lytic proteins (Table 1). Many of the proteins encoded by KSHV lytic genes also have pro-growth or transforming abilities. Major functions of KSHV lytic proteins include cellular proliferation and evading the host’s immune response. The immune functions targeted by viral proteins include IFN production, interferon regulatory factor (IRF) activation, complement activation, inflammasome, and chemokine activation (Figure 3).

**Table 1:** KSHV lytic proteins involved in immune modulation and pathogenesis.

KSHV genes	KSHV proteins	Function	References
K1	Variable ITAM-Containing Protein (VIP)	Type I transmembrane signaling protein containing a functional immunoreceptor tyrosine-based activation motif. Regulate membrane transport in B cells.	[203]
K2	Viral Interleukin-6 (vIL-6)	Homologues of cellular IL-6. Activate JAK/STAT, MAPK, and PI3K/Akt signaling pathways to regulate B-cell proliferation.	[51,204]
K3/K5	Modulator of immune recognition (MIR1/MIR2)	Viral E3 ligases capable of ubiquitinating MHC-I, ICAM-1, B7-2, Tetherin (CD317/BST2), DC-SIGN, and DC-SIGNR.	[205,206]
K4/K4.1/K6	Viral CC-Chemokine Ligands (vCCLs)	Homologues of cellular chemokines: viral CC-chemokine ligand 1 vCCL1 (vMIP1), vCCL2 (vMIP2), and vCCL3 (vMIP3), respectively. Blocks signaling through chemokine receptors.	[207,208]
K7	Viral Inhibitor of Apoptosis (vIAP)	Interact with cellular proteins PLIC1, caspase 3/Bcl-2, CAML, Vps34, and promote cell survival during lytic replication.	[209,210]
K9/K10/K11	KSHV interferon regulatory factors (vIRF-1, vIRF-2, vIRF-3 and vIRF-4)	Homologues of cellular interferon: Inhibitor of IFN1, p53, NFκB RelA, and p300.	[211,212]
K14	vOX2 or vCD200	Homologues of cellular OX2. A negative regulator of inflammatory signaling and surface glycoproteins.	[213,214]
K15	Viral membrane protein	Regulation of cellular signaling to induce various pro-survival and paracrine-mediated pro- angiogenic cellular cytokines and chemokines, including IL6, IL8, IL-1a/b, CXCL3, and Cox2.	[215,216]
ORF4	KSHV complement Control protein (KCP)	Homologue to cellular RCA. Regulate complement activation by increasing the decay of the classical C3 convertase.	[217,218,219]
ORF45	ORF45	Inhibit type1 IFN induction by sequestering the cellular interferon regulatory factor-7 to cytoplasm.	[220,221]
ORF63	ORF63	Homologue to cellular inflammasome complex NLRP1.	[222]
ORF64	Viral deubiquitinase	A non specific deubiquitinase, shown to deubiquitinate RIG-I to suppress RIG-I-mediated activation of the IFNb.	[223]
ORF74	Viral G-protein-coupled receptor (vGPCR)	Homologue of cellular IL-8 receptor. vGPCR induce secretion of proinflammatory cytokines and angiogenic growth factors.	[200,224]
ORF75	ORF75	A viral effector for the degradation of ND10 proteins.	[225,226]
PAN RNA	Polyadenylated Nuclear RNA	Modulator of viral gene expression.	[227,228,229,230]

KSHV employs diverse mechanisms for controlling both IFN production and signaling as IFN is a potent antiviral defense that is critical for KSHV persistence [231]. The genomic region encompassing ORFs K9 to K11 encodes KSHV vIRFs 1-4 [232]. vIRF1 can bind to and disrupt the transcriptional activities of IRF1, IRF3, and IRF7 [211,212]. Additionally, vIRFs 1, 3, and 4 have been shown to inhibit p53 activity via, either direct binding to the tumor suppressor (vIRF-1 and vIRF-3), or through association with ATM kinase or via stabilization of MDM2, which induces ubiquitination and proteasomal degradation of p53 [233,234]. Similarly, KSHV viral interleukin-6 (vIL6), encoded by ORF K2, shares many functional characteristics with human IL6 and, as a result, the viral cytokine can activate gp130 and downstream signaling pathways, including the JAK/STAT, MAPK, and PI3K/Akt pathways [204,235]. These pathways regulate a variety of transcription factors and response elements (RE), such as the STAT1/3 and STAT5 IL6 RE, C/EBP, and c-jun promoter IL6 RE (JRE-IL-6) [236]. A viral homologue of the cellular angiogenic IL-8 receptor [224], vGPCR has been shown to activate a number of crucial signaling pathways, including PLC, PKC, MAPK, PI3K/Akt/mTOR, and NFκB [237]. Downstream signaling from these pathways activates the AP1, NFAT, NF-kB, HIF-1a, and CREB transcription factors, which, in turn, contribute to vGPCR-mediated production of pro-inflammatory cytokines and chemokines [237].

Many of the KSHV K-gene encoded lytic proteins have also been shown to modulate KSHV infection and pathogenesis. Multifunctional transmembrane glycoprotein K1 encoded by the first ORF of KSHV can constitutively activate multiple pro-growth signaling pathways in KSHV-infected cells. [238]. Oligomerization of K1 trigger auto-phosphorylation of ITAM and activate various Src homology 2 (SH2) containing signaling proteins, including PI3K (p85)/Akt, PLCg, Vav, Syk, Lyn, RasGAP, and Grb2 [239,240]. Similarly, it has been shown that K15-activated cellular signaling pathways induce the transcription of a number of cellular cytokines and chemokines, including IL6, IL8, CCL20, CCL2, CXCL3, IL-1a/b, and Cox2 [215,216]. Additionally, KSHV K7 or viral inhibitor of apoptosis (vIAP), is a homologue of cellular Bcl-2 proteins and contains a putative mitochondrial-targeting signal and localizes to mitochondria and ER [210,241]. It has been reported that K7/vIAP inhibits caspase 3 activity by interacting with cellular Bcl-2 via its BIR (baculovirus IAP repeat) [210]. Furthermore, KSHV K3 and K5 (also called modulator of the immune recognition (MIR) 1 and 2, respectively) are viral E3 ligases capable of ubiquitinating the MHC-I cytoplasmic tail to trigger internalization and proteasomal degradation of the MHC-I complex [205,242,243]. K3 and K5 proteins also have been shown to downregulate both C-type lectins, DC-SIGN, and DC-SIGNR by ubiquitin mediated degradation [206]. Similarly, KSHV K6, K4, and K4.1 encode three homologues of cellular chemokines: viral CC-chemokine ligand 1 vCCL1(vMIP1), vCCL2 (vMIP2), and vCCL3 (vMIP3), respectively [154,244,245]. Apart from immune evasion properties, v-chemokines also have been shown to promote angiogenesis through the induction of VEGF [246,247]. KSHV-encoded early lytic protein K14 is another negative regulator of inflammatory signaling and surface glycoprotein (vOX2). K14 shows significant homology with OX2 or CD200, a member of the immunoglobulin superfamily that is broadly distributed on the cell surface [90]. vCD200 promotes the secretion of proinflammatory cytokines on stimulation of monocytes, macrophages, and DCs through a direct interaction with cellular CD200R, inhibiting myeloid cell activation and reducing Th1-cell-associated cytokine production [214,248].

Furthermore, KSHV-ORF4-encoded inhibitor of the complement system, designated as KSHV complement Control Protein (KCP) [217,249,250], regulates complement by increasing the decay of the classical C3 convertase and acting as cofactors for the inactivation of C3b and C4b, components of the C3 and C5 convertases [251,252]. Similarly, KSHV encoded ORF45, an immediate early gene product, plays a crucial role in lytic replication [253]. ORF45 has been shown to inhibit type1 IFN induction upon infection by sequestering the cellular interferon regulatory factor-7 (IRF-7) to the cytoplasm [220,221]. It has been shown that ORF45 can also regulate eIF4B phosphorylation in an mTOR and MAPK independent manner. Additionally, the ORF45 protein is also involved in the transport of the viral capsid-tegument complexes along the microtubule filaments [254].

It has been also been shown that KSHV encoded tegument protein, ORF75 is an essential protein as a new viral effector [255] for the degradation of ND10 proteins, thereby regulating lytic replication and KSHV infection [225]. In addition, the ORF75 also has been shown to induce the degradation of ATRX and relocalization of Daxx, as well as be involved in NF-kB coactivation with KSHV K13/vFLIP [225,256]. Similarly, KSHV encoded ORF64 is a deubiquitinase that non-specifically targets K48 or K63 ubiquitination. It has been shown that KSHV ORF64 is capable of deubiquitinating RIG-I to suppress RIG-I-mediated activation of the IFNb promoter [223]. Studies showed that KSHV ORF63 has homology to parts of cellular inflammasome complex NLRP1 [222,257]. This ORF63 function seems to be critical for supporting viral gene expression and genome replication, as well as suppressing IL-1b production [222,258]. Additionally, KSHV encoded structural PAN RNA has been also shown as a multifunctional transcript that can globally control viral and cellular gene expression during lytic reactivation [259] through direct interaction with chromatin modifying complexes, such as components of PRC2 [228,229]. PAN RNA interacts with demethylases, UTX, and JMJD3 to modify the suppressive H3K27me3 mark within the KSHV genome [260]. Moreover, PAN RNA expression decreased the expression of interferon γ, interleukin 18, interferon α16, and RNase L [229].

## 6. Conclusions

Kaposi’s sarcoma associated herpesvirus (KSHV) modulates various cellular pathways by which it is able to establish and maintain persistent infection in the host to initiate tumorigenesis. Several of these latent viral and lytic proteins are known to transform host cells, linking KSHV with the development of severe human malignancies. These virus-induced cancers pose a large threat to global public health, specifically in areas that are still struggling with malignancies associated with HIV-AIDS with limited treatment options. Over the years, tremendous progress has been made in elucidating the molecular mechanisms of KSHV latency and lytic replication. Nonetheless, there are still vast aspects of viral infection and transformation that are not well explored. With the help of rapid advancements in modern technology, it is presumed that a thorough knowledge of the KSHV life cycle will be achieved over the next few years. Further understanding of the unique mechanisms that KSHV adopts for the establishment of successful lifetime persistence in the infected host will eventually pave the way for novel therapeutic approaches for the treatment of KSHV diseases.
